# The Effect of Light Exposure at Night (LAN) on Carcinogenesis via Decreased Nocturnal Melatonin Synthesis

**DOI:** 10.3390/molecules23061308

**Published:** 2018-05-29

**Authors:** Aldo Giudice, Anna Crispo, Maria Grimaldi, Andrea Polo, Sabrina Bimonte, Mario Capunzo, Alfonso Amore, Giovanni D’Arena, Pellegrino Cerino, Alfredo Budillon, Gerardo Botti, Susan Costantini, Maurizio Montella

**Affiliations:** 1 Epidemiology Unit, IRCCS Istituto Nazionale Tumori “Fondazione G. Pascale”, 80131 Napoli, Italy; a.crispo@istitutotumori.na.it (A.C.); m.grimaldi@istitutotumori.na.it (M.G.); 2 Experimental Pharmacology Unit, IRCCS Istituto Nazionale Tumori “Fondazione G. Pascale”, 80131 Napoli, Italy; a.polo@istitutotumori.na.it (A.P.); a.budillon@istitutotumori.na.it (A.B.); 3 Division of Anesthesia and Pain Medicine, IRCCS Istituto Nazionale Tumori “Fondazione G. Pascale”, 80131 Napoli, Italy; s.bimonte@istitutotumori.na.it; 4 Department of Medicine Surgery and Dentistry, University of Salerno, Baronissi, 84081 Salerno, Italy; mcapunzo@unisa.it; 5 Abdominal Surgical Oncology and Hepatobiliary Unit, IRCCS Istituto Nazionale Tumori “Fondazione G. Pascale”, 80131 Napoli, Italy; a.amore@istitutotumori.na.it; 6 Department of Hematology and Stem Cell Transplantation Unit, IRCCS, Cancer Referral Center of Basilicata, 85028 Rionero in Vulture, Italy; giovannidarena@libero.it; 7 Istituto Zooprofilattico Sperimentale del Mezzogiorno (IZSM), 80055 Portici, Napoli, Italy; aziendacerino@gmail.com; 8 Pathology Unit, IRCCS Istituto Nazionale Tumori “Fondazione G. Pascale”, 80131 Napoli, Italy; g.botti@istitutotumori.na.it

**Keywords:** melatonin, circadian rhythms, cancer

## Abstract

In mammals, a master clock is located within the suprachiasmatic nucleus (SCN) of the hypothalamus, a region that receives input from the retina that is transmitted by the retinohypothalamic tract. The SCN controls the nocturnal synthesis of melatonin by the pineal gland that can influence the activity of the clock’s genes and be involved in the inhibition of cancer development. On the other hand, in the literature, some papers highlight that artificial light exposure at night (LAN)-induced circadian disruptions promote cancer. In the present review, we summarize the potential mechanisms by which LAN-evoked disruption of the nocturnal increase in melatonin synthesis counteracts its preventive action on human cancer development and progression. In detail, we discuss: (i) the Warburg effect related to tumor metabolism modification; (ii) genomic instability associated with L1 activity; and (iii) regulation of immunity, including regulatory T cell (Treg) regulation and activity. A better understanding of these processes could significantly contribute to new treatment and prevention strategies against hormone-related cancer types.

## 1. Introduction

**Figure 1:**
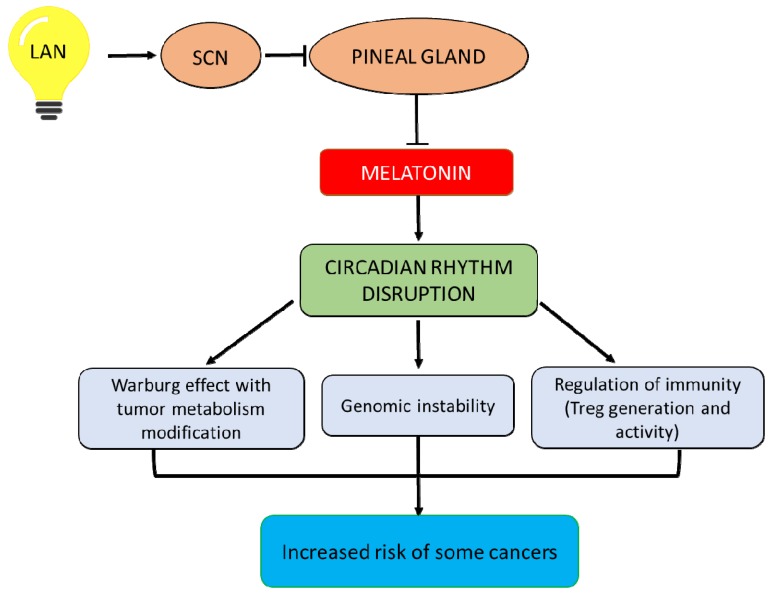
Artificial light exposure at night (LAN) influences the master clock present in the suprachiasmatic nucleus (SCN) of the hypothalamus. This leads not only to a decrease in nocturnal melatonin synthesis in the pineal gland but also to circadian rhythms disruption. These events can increase the risk for several melatonin-sensitive human cancers mainly through several mechanisms, including the Warburg effect related to tumor metabolism modification, genomic instability, and modification of immunity via Treg cells.

The master clock located in the suprachiasmatic nucleus (SCN) of the hypothalamus is able to drive the circadian rhythms observed in a majority of functions, such as hormone production, immune activity, and blood pressure [1]. Apart from the SCN, there are also peripheral clocks located in other tissues or cells [2]. Desynchronization of the circadian system and the environment can induce disruption of circadian coordination and different health problems [3,4]. For example, disruption of circadian rhythms may be associated with hormone imbalance, sleep disorders, coronary heart attacks, depression, and cancer proneness [5]. Studies on animal models and on human cancer samples, such as tissues and biological fluids, show that an alteration of circadian rhythms can increase the risk of cancer development and progression [5]. Specifically, circadian disruption leads to aberrant epigenetic modifications, such as hypermethylation, which play an important role in the transformation of different human normal cells into cancer cells [6]. However, several environmental factors, such as irregular diet (meals) or night-shift work, that are correlated with exposure to artificial light at night, are able to disrupt circadian rhythms mostly by altering nocturnal melatonin biosynthesis [4]. The focus of this review is to examine the impact of circadian disruption due to environmental factors on the control of tumor growth. In particular, we describe the potential mechanisms by which light exposure at night (LAN) can increase cancer risk considering also the decision of the World Health Organization (2007) that has recognized LAN as a probable carcinogen. In fact, LAN can block the pineal gland and the related nocturnal production of melatonin in individuals, such as shift workers, that are regularly subjected to LAN, and induce circadian rhythms disruption. These events can increase risk for several melatonin-sensitive human cancers mainly through several mechanisms, including the Warburg effect related to tumor metabolism modification, genomic instability, and modification of immunity via Treg cells (see Figure 1).

## 2. The Mammalian Biological Clock

The master clock, also known as the central circadian clock, is located within the SCN of the anterior hypothalamus in mammals. Considering that light is the most potent regulator for the SCN, it is important to underline that it passes into a mammal’s system through the retina and is collected by rod cells and cone cells before being transmitted to the retinal ganglion cells. These cells use the photopigment melanopsin to absorb the light energy that, then, arrives to the neurons through the retino-hypothalamic tract (RHT). On the other hand, the SCN can integrate these signals by melatonin [7] and the central circadian clock can support the neurons in resisting shifts to sporadic stimuli. Recently, circadian rhythms have been found in extra-SCN brain regions and in many peripheral tissues, including oral mucosa, liver, skin samples, bone marrow, and peripheral blood mononuclear cells (PBMCs). The expression of clock genes in peripheral tissues is associated with important tissue functions, such as glucocorticoid production, regulation of the cell-cycle, fatty acid metabolism and cytokine production by NK cells, B cell maturation, and adipogenesis in cardiomyocytes and bone formation [1]. The central circadian clock in the SCN regulates the peripheral clocks through neuroendocrine systems and the autonomic nervous system (ANS) [8].

## 3. Molecular Mechanism of the Mammalian Biological Clock

In the last few years, many authors have focused on the study of circadian rhythms that are present in organisms from bacteria to mammals [9]. A molecular clock is composed of regulatory transcription–translation loops underlining the reciprocal interrelationships between particular genes. The following nine mammalian circadian genes are known: brain-muscle-Arnt-like1 (BMAL1), Casein kinase 1ε (CK1ε), Circadian locomoter output cycles protein kaput (CLOCK), Cryptochrome1 (CRY1), Cryptochrome2 (CRY2), Period1 (PER1), Period2 (PER2), Period3 (PER3), and Timless (TIM). Some key circadian regulators contain basic helix-loop-helix (bHLH) or Per-Arnt-Sim (PAS) domains and act as transcription factors in interactions between proteins or a protein and DNA forming a self-sustained transcriptional feedback loop. In fact, changes in cellular localization or concentration or delays between translation and transcription induce a 24 h cycle [10]. In the mouse, CLOCK represents the first identified clock gene, capable of inducing CLOCK-BMAL1 heterodimers formation, to bind to E-box (5′-CACGTG-3′) in the promoters of Rev-Erbα and CRY/PER and to activate transcription when a circadian day starts. In turn, CRY1, CRY2, PER1, PER2, and PER3 in the cytoplasm induce the formation of stable CRY-CKIε-PER complexes before entering the nucleus. When CRY1 is in the nucleus, it disrupts the transcriptional complex associated with BMAL1-CLOCK, and inhibits BMAL1, Rev-Erbα, and PER transcription [11,12]. Recently, it has been demonstrated that BMAL1 can form some dimers with neuronal PAS domain protein 2 (NPAS2) and sustain rhythmicity by activating transcription [13]. Few data are available about the control of CLOCK and BMAL1 expression. However, some studies showed that BMAL1 transcription is negatively regulated by Rev-Erbα in the SCN and in the liver and positively regulated by PER, CRY, and retinoic acid receptor-related orphan receptor α (RORα) [11,14,15]. On the other hand, Rev-Erbα plays an important role also in the regulation of CLOCK transcription. However, Rev-Erbα is not necessary for circadian rhythm generation even if it is implicated in the phase-shifting properties of the clock and in the period length. In addition, to simple transcriptional feedback loops, other events, such as the control of degradation, nuclear translocation, and protein phosphorylation, are implicated in circadian rhythm generation. For example, CKIε activates BMAL1 transcription and regulates the phosphorylation of PER and CRY by enhancing their degradation and instability [16]. Interestingly, these studies demonstrate that the control and maintenance of a circadian period is due to different factors correlated to post-translational modification, protein synthesis and degradation, and dimer formation.

## 4. Alteration of Circadian Rhythm and Carcinogenesis

Many papers show that the disruption of circadian rhythms is associated with various forms of cancer, including the hormone-related cancers that are frequent in shift workers because they develop abnormal work hours capable of inducing circadian rhythm disruption [17,18,19,20,21]. According to this notion, Franzese and Nigri (2007) reported that individuals who work at night, for example nurses, present decreased melatonin levels and dysregulated hormone profiles and a higher risk of breast cancer development [18]. Other authors have demonstrated that 95% of breast cancers show dysregulated levels of PER1 and PER2 that are not due to genetic mutations but to methylation of these two genes [19,20]. However, mice studies have demonstrated that the disruption of circadian rhythms can be involved also in the development and progression of pancreatic adenocarcinoma or osteosarcoma [22]. Also Sephton et al. (2000) reported that patients with metastatic breast cancer had lower survival when they present flattened or abnormal levels of diurnal salivary cortisol rhythms [23]. In turn, patients with metastatic colorectal cancer show longer survival when 24 h rest/activity rhythms are marked and not disrupted [24]. A higher risk of acute myeloid leukaemia was found, in 1996, in men working for Air Canada [25]. Circadian rhythm and sleep control disturbance is also associated with lymphoma development and progression [26,27]. In fact, PER2-deficient mice present decreased transcription of genes that are implicated in cell cycle regulation, salivary gland hyperplasia, and high risk of lymphoma initiation. In detail, Fu et al. (2002) performed a transcriptome analysis evidencing that many important cell-cycle genes are regulated in a circadian fashion and control various points of cell division. Among these genes, there are MYC involved in G0/G1 transition, cyclin-D1 involved in G1/S transition, and WEE1 involved in G2/M transition [27]. If this idea is true, then it could provide insight into human proliferative diseases, such as cancer.

Overall, these studies demonstrate an important role of circadian rhythms in cancer even if few data are available in the literature about the molecular mechanisms through which these factors are able to induce cancer development and progression. For this reason, the correlation between melatonin and circadian rhythms represents an interesting topic.

## 5. Circadian Rhythms and Melatonin in the Control of Tumor Growth

Melatonin is a molecule, known since 1958, whose presence and function is phylogenetically well-recognized in a variety of species from unicellular to vertebrate organisms. It is also a pleiotropic compound with a large repertoire of actions that operate in a diverse number of biological contexts. In mammals, the nocturnal production of melatonin is regulated by the circadian clock located in the SCN of the hypothalamus. In particular, melatonin is synthesized in the pineal gland starting from l-tryptophan by four steps: (i) Hydrolyzation of l-tryptophan by tryptophan hydroxylase that produces 5-hydroxytryptophan (5-HTP); (ii) decarboxylation of 5-HTP by pyridoxal phosphate and 5-hydroxytryptophan decarboxylase that produces serotonin; (iii) *N*-acetylation of serotonin by arylalkylamine *N*-acetyltransferase (AANAT) that produces *N*-acetylserotonin; and (iv) conversion of *N*-acetylserotonin to melatonin by hydroxyindole-*O*-methyltransferase (HIOMT). The regulation of melatonin levels is given by norepinephrine, which in turn induces the increase of cAMP concentration and the activation of cAMP-dependent protein kinase A (PKA) [28]. However, AANAT is considered the key enzyme in the regulation of all of the mechanisms which lead to melatonin production. In particular, both elevated cAMP levels and PKA activation are indispensable to the stimulation of AANAT and, hence, melatonin production in all mammals [28]. The activity of AANAT displays marked day/night variations (>100-fold nocturnal increase) whereas that of HIOMT only increases by about 50% at night. Therefore, AANAT is considered the “rate-limiting” enzyme for melatonin biosynthesis in the pineal gland [29].

Rat hepatoma, human and rat breast cancer, and human leiomyosarcoma cancer are examples of cancers that are responsive to the inhibitory actions of melatonin [30,31,32,33,34]. Some authors have demonstrated that melatonin: (i) increases CLOCK and PER2 levels and decreases those of BMAL1 in prostate cancer cells [35]; (ii) decreases RORα-mediated BMAL1 expression in breast cancer cells [36]; (iii) has anti-oxidant properties because it scavenges the free radicals and induces an increase of glutathione peroxidase (GSH-Px) and superoxide dismutase (SOD) levels [37]; (iv) reduces metastases in murine breast cancer [38] and human breast cancer [39]; and (v) inhibits breast cancer cell invasion by downregulating the p38 pathway and by blocking MMP-2 and MMP-9 activity [39]. On the other hand, it is well-known that circadian genes regulate cell proliferation and apoptosis and, hence, the expression of caspases or genes involved in the cell cycle or in cancer suppression [1]. Considering these features, many papers have demonstrated that disruption of the circadian clock increases the risk to develop human cancers. In fact, loss of the PER2 gene can damage p53-mediated apoptosis, activate c-Myc signaling pathways, and induce an increase of cell proliferation and genomic instability [1].

Taken together, these data indicate that the circadian disruption associated with dysregulation of melatonin production can modify the synchronization of peripheral clocks and, in turn, affect cancer cell proliferation cycles [40].

## 6. Tumor Growth Inhibition through the Warburg Effect and Metabolism of Linoleic Acids

In the last few years, several authors have evidenced that there is a connection between neuro-immunomodulatory control loops and cancer initiation and progression. Various findings in animals and humans have demonstrated that light can suppress melatonin synthesis and can increase the levels of cortisol, estrogens, and androgens [41]. Consistent with this notion, Stevens showed that, in industrialized societies, light exposure at night induces a block of the pineal gland, a block of the nocturnal production of melatonin, an increase of the estrogen levels in postmenopausal women, and, hence, a higher risk to develop breast cancer [42]. Another important aspect is that melatonin’s cancer inhibition in vivo involves linoleic acid (LA), the most prevalent essential omega-6 polyunsaturated fatty acid (PUFA) that presents with high levels in the Western diet [43]. In fact, the elimination of the nocturnal melatonin signal by LAN is able to enhance nocturnal LA uptake and its conversion to 13-hydroxyoctadecadienoic acid (13-HODE) and increase breast cancer risk, which represents a significant public health problem [44]. These factors were used to explain the breast cancer development in some women who had worked night shifts for many years [44]. Experimental evidence in both human cancer xenografts and rat hepatoma 7288CTC showed that LA uptake and its metabolism are blocked through the decrease of cAMP levels [45,46,47,48]. In fact, Blask et al. (1999) indicated that, during light exposure, when circulating melatonin levels are very low, cancer LA uptake is strongly elevated due to high levels of c-AMP that are capable of increasing FATP (transport proteins) activity mainly through their phosphorylation. Intracellular LA is rapidly metabolized to 13-HODE by an EGF-activated lipoxygenase [45]. In many tumors, increased 13-HODE levels positively stimulate EGF and IGF-1 receptors to enhance ERK1/2 and AKT phosphorylation, leading to enhanced EGF and IGF-1-dependent cell mitogenesis and survival [49]. Blask al. (2014) also demonstrated that high levels of 13-HODE in cancer cells can induce AKT activation, drive the Warburg effect, and hence convert the normal cellular metabolism from oxidative phosphorylation to aerobic glycolysis with a great oxygen supply [50] (see Figure 1). It is possible that the AKT oncogene, present in many cancers, is sufficient to stimulate continued transformed cells growth and survival with a relative increase of glucose use without consuming oxygen [51]. A higher rate of glucose uptake and its metabolism to lactate via glycolysis might ensure a constant supply of the molecular intermediates essential for protein and nucleic acid synthesis to support proliferative activity of cancer cells. Finally, the production of NADPH through the pentose phosphate cycle can induce a decrease of oxidative stress in many types of malignant cells [52]. Blask et al. [50] also demonstrated that during a dark phase, when circulating melatonin levels are high, melatonin suppresses the cancer production of 13-HODE and blocks LA uptake by cAMP action and by MT1 receptors. As a consequence, suppression of 13-HODE production and subsequent attenuation of Akt signaling might indirectly inhibit the Warburg effect, cell proliferation, and tumor growth [50]. Moreover, other papers have reported that: (i) the melatonin levels are lower in prostate cancer patients than in normal men [53]; and (ii) the melatonin interacts with the androgen receptor and modulates the cell growth in prostate cancer patients [54]. Additionally, Dauchy et al. (2015) demonstrated that daytime exposure of rats to blue light significantly increased nocturnal melatonin levels, resulting in a strong suppression of cancer growth by a marked reduction of cancer cAMP levels, LA uptake, 13-HODE formation, and the Warburg effect [55]. In contrast to these results, colorectal cancer mucosa presented lower levels of 13-HODE and 15-lipoxygenase-1 that, in turn, are able to inhibit colon cancer growth in vivo [56,57,58]. Therefore, these authors underlined that 13-HODE’s ability to induce cancer growth is “tissue-specific”.

## 7. Receptor-Mediated Effect of Melatonin on the Control of Genomic Instability 

Another possible mechanism that can mediate cancer inhibition by melatonin is the regulation of genomic instability associated with retrotransposons, such as Long Interspersed Element-1 (L1), that are present not only in all mammals and humans but can be also found in plants, protozoa, and fungi [59,60]. Several papers have investigated the extent of LINE-1’s contribution to tumorigenesis even if its mechanistic relevance remains unknown. For example, some studies indicate that: (i) L1 is not expressed or presents with very low levels in normal adult human tissues and early stages of tumorigenesis [60,61,62]; (ii) somatic L1 insertions can inactivate the tumor suppressor APC gene in colon cancer cells [63]; (iii) L1 expression and retrotransposition are inhibited by multiple mechanisms, including the methylation status of L1 [64]; (iv) the frequent hypomethylation of chromatin in tumor cells is the main mechanism responsible for L1 mobility [65]; and (v) L1 contributes to cancer development by inducing hTERT and maintaining telomeres in telomerase-positive tumor cells [66]. However, L1 expression can be regulated by acetylation and methylation of histones [67], noncoding RNAs, such as Piwi-interacting RNAs, siRNAs, and miRNAs [65,68], and self-regulation by the L1 antisense promoter [69]. In 2014–2015, a research group suggested that melatonin, through an MT1-receptor-mediated action, can downregulate L1 and inhibit its mobilization, consequently leading to a decrease in L1-associated genomic instability in human cancer cell lines [70,71]. Other studies by Kang and Sancar (2009) also indicate that normal melatonin signal suppression may indirectly enhance L1-induced genomic instability and tumorigenesis through the circadian disruption of core clock proteins operating in peripheral tissues capable of interacting with cellular DNA repair proteins usually involved in the suppression of L1 retrotransposition [72]. Further, the suppression of melatonin/melatonin receptor signaling is able to modify different pathways necessary for cellular proliferation and apoptosis. For instance, Santoro and Strano (2013) reported that melatonin can activate the MT1 and MT2 receptors, induce a DNA damage response, and preserve genome integrity. In addition, they also suggested that, in the absence of both receptors, melatonin is not able to decrease cell proliferation and to block DNA damage [73]. Further studies, recently published by Wylie A et al. (2016), also demonstrated that p53, by genetically interacting with components of piRNA (piwi-interacting RNA), might suppress the mobility of retrotransposons that are well-documented piRNA targets. Accordingly, human p53 mutants or p53 loss was strongly associated with transposon mobility compared to normal counterparts. All these data suggest the possibility that p53 may contribute to oncogenesis suppression not only by preventing DNA damage but also by restricting the movement of mobile elements [74]. In addition, studies by Mao L et al. (2012) also reported that melatonin activates glycogen synthase kinase 3β (GSK3β) by inhibiting serine-threonine kinase Akt phosphorylation, by inducing β-catenin degradation, and by inhibiting epithelial-to-mesenchymal transition, a fundamental process underlying cancer metastasis [75]. As a consequence, nocturnal melatonin signal inhibition via exposure to light at night could increase genomic instability and the carcinogenesis process. Another important aspect underlined by Coon SL et al. (2012) is that some long non-coding RNAs (IncRNAs) follow the circadian clock and are able to modify melatonin biosynthesis [76]. Hence, the dysfunction of the circadian machinery due to LAN may alter melatonin production and consequently promote carcinogenesis not only by increasing nocturnal tumor uptake of LA but also by promoting L1-retrotransposon-associated genomic instability and also by changing the level and activity of certain IncRNAs [76].

Certainly, it is important to underline that the current literature related to L1’s connection to LAN- and circadian-disruption-associated risk of cancer is very limited and speculative and further studies will be necessary to understand this correlation in more detail.

## 8. Immunoregulation by Melatonin

It is well-known that cancers evade the immune system by producing factors able to block immune TH1 response and activate TH2 response [77,78]. Melatonin can suppress this process by increasing the levels of IL2 that favor T cell differentiation and increase IFN-g production [79]. Considering that melatonin is able to suppress apoptosis in normal cells and to induce apoptosis in cancer cells, it has been indicated to be useful in cancer treatment [80]. Recent studies by Bollinger et al. (2009) demonstrated that nTregs (CD4^+^ and CD25^+^, natural regulatory T cells) follow circadian rhythms and increase their levels during night as well as that of melatonin. Hence, sleep deprivation blocks the normal function of nTreg [81] (see Figure 1). Recent studies indicate that melatonin decreases the levels of Tregs and Foxp3 and blocks gastric cancer progression even if no direct effects were observed on lymphocytes cultured “in vitro” [82,83]. Therefore, the authors suggested that: (i) melatonin may inhibit Treg cell production by such indirect mechanisms as, for example, the inhibition of macrophage activity, which is involved in Treg cell stimulation [84,85]; and (ii) melatonin can increase chemotherapy efficacy through the alteration of the macrophage system and the related suppression of Treg cell production [86]. Recently, Kassayova M et al. (2016) demonstrated that Lactobacillus plantarum LS/07 and inulin exert antiproliferative and immunomodulatory activities in rat mammary carcinogenesis and that these activities are significantly amplified by melatonin co-administration [87]. In particular, melatonin co-administration markedly increased both tumor infiltration by Treg cell production and tumoral Ki-67 expression. These results demonstrated for the first time that melatonin is capable of increasing the local immune response induced by a combination of a probiotic and a prebiotic in mammary tumor tissue [87]. In addition, such findings also indicate that the role of Treg cells in cancer development remains controversial. In fact, despite numerous reports indicating a positive association between Tregs and progression of solid tumors [88], an increasing number of studies also suggest that tumor-infiltrating Tregs are an index of a good prognosis [89,90]. Moreover, in Estrogen Responsive (ER)-negative breast cancer patients, a high degree of concurrent cancer infiltration by Tcells and Tregs is correlated with robust anticancer immunity and a good clinical outcome [91]. Notably, transforming growth factor-beta (TGF-β) is a growth factor capable of modulating Tcell proliferation and differentiation [92] and of inducing Foxp3 expression [93]. Studies by Proietti et al. (2011) suggest that melatonin alone or with vitamin D3 in early stages of mammary carcinogenesis can inhibit cell growth and activate the TGF-β1 pathway promoting apoptosis [94]. In addition, it is important to remember that the proinflammatory cytokine IL-6 has an important role in estrogen-dependent tumor growth and inhibits Treg cell production [95]. Therefore, it is not excluded that melatonin administration alone or with vitamin D3 and a probiotic and a prebiotic can inhibit the estrogen-dependent breast carcinogenesis process by promoting peripheral Tregs differentiation in mammary tumor tissue by activation of the TGF-1β pathway and concomitantly by inhibiting the NF-kB/COX-2/IL-6 pathway as well as the development of Tregs [93,94,96]. There is also evidence that the carcinogenesis process is often associated with several neuroendocrine disorders. Among them, the most important are: (i) the disappearance of cortisol circadian rhythms in many malignant tumors; (ii) abnormally high levels of prolactin; and, in particular, (iii) pineal endocrine deficiency or a progressive decline in the nocturnal melatonin production that represents the main and the most investigated cancer-progression-related neuroendocrine alteration [97]. Notably, at present, melatonin represents the only molecule existing in nature that is potentially capable of suppressing the overall phases of cancer development, progression, invasion, and neoangiogenesis [98,99,100,101,102]. Certainly, progressive decreasing of melatonin at night could significantly contribute to cancer development and neoplastic progression [100]. Notably, many studies suggest that tumor production of indoleamine-2,3-dioxygenase (IDO) may progressively reduce melatonin blood levels by inducing a depletion of the amino acid tryptophan (Trp) [103]. Such tryptophan deficiency and/or the accumulation of Trp catabolites, such as kinurenine (Kyn), 3-hydroxykynurenine, and 3-hydroxyanthranilic acid, can influence both melatonin synthesis and the anticancer immune response since tryptofan depletion promotes immune tolerance (or suppression of the anticancer response) through direct inhibition of TH1-lymphocyte functions and proliferation or by stimulating regulatory T lymphocyte activation (Tregs) [103,104]. Another interesting study recently published by Ren W et al. (2017) also suggests that melatonin does not alter Foxp3+ cell frequency in normal conditions, but only in inflammatory diseases, and it decreases significantly the number of Tregs cells in immunosuppressive conditions [105]. Other reports also indicate that elevated levels of glucocorticoids may lead to impaired melatonin production by the pineal gland and subsequent immunosuppression capable of promoting carcinogenesis or malignant transformation [106]. Further, in malignant tumors, severe hypoxia was often associated with a decreased differentiation of CD4+ effector T cells and an increased number of Treg cells. Clambey et al. (2012) suggest that hypoxia inducible factor 1α (HIF-1α) regulates CD4+ T-cell functions in hypoxic conditions. Specifically, these authors demonstrated that hypoxia increases the abundance and function of Tregs by stimulating Foxp3 expression without acting on the pre-existing Tregs and that HIF-1α suppresses inflammation and tissue damage in conditions of reduced oxygen availability [107]. Other authors also reported that severe hypoxia in a mouse model with colitis-associated colon cancer increases not only the Tregs’ abundance but also the expression of antinflammatory and immunosuppressive cytokine IL-10, which directly inhibits the CD4^+^ T-cells’ function [108,109]. Since it seems that HIF-1α increases Treg cells production not only in inflammatory pathologies, such as rheumatoid arthritis or inflammatory bowel disease, but also in the tumor microenvironment, the development of an anticancer strategy targeting HIF-1 activity represents an interesting scientific topic. In fact, studies by Park et al. (2009) suggested that pharmacological concentrations of melatonin can inhibit growth factors (IGF-1, insulin), and induce HIF-1 production rather than its proteasomal degradation [110]. Finally, melatonin also suppresses tumor angiogenesis by inhibiting not only HIF-1α accumulation through suppression of the melatonin nuclear receptor RZR/RORγ and the SUMO-specific protease 1 (SENP1) signaling pathway but also VEGF production in human gastric cancer cells under hypoxia [111].

Overall, these data collectively highlight that melatonin administration can correct several cancer-related neuroendocrine disorders involved in the control of anticancer immunity by significantly improving the prognosis of cancer patients.

## 9. Conclusions

Several epidemiologic and experimental observations have shown that circadian rhythms regulate many physiological processes, such as sleep, hormone production, immune activity, and cell proliferation and apoptosis. As a consequence, circadian coordination disruption increases the risk for a variety of pathologies, such as psychiatry disorders and metabolic alterations, and promotes cancer development and progression [1]. On the other hand, melatonin is a pineal hormone that maintains the daily clock. Its nocturnal synthesis is inhibited by light exposure at night (LAN) [112]. Consequently, the blocking of circadian melatonin production can increase the risk of cancer development [41,42,53]. In support of this notion, melatonin-rich sera collected at night compared to melatonin-depleted sera collected after LAN significantly suppressed the proliferative activity of human breast cancer xenografts in perfused rats [31]. There are several mechanisms (Figure 1) by which LAN might increase risk for several human cancer types by its ability to inhibit melatonin production at night: (i) nocturnal tumor uptake of dietary linoleic acid and subsequent production of 13-HODE; (ii) genomic instability associated with retrotransposons, such as L1, capable of activating oncogenes and inactivating tumor suppressor genes primarily through insertional mutagenesis and suppression of the expression of LINE-1; and (iii) regulation of Treg cells production.

Therefore, we can suggest that new therapeutic strategies are necessary to protect the integrity of the circadian rhythm melatonin signal. As an example, the association of circadian-timed physiologic melatonin supplementation with modifications in nocturnal lipid intake could represent a useful approach for blocking cancer initiation and/or for decreasing cancer progression. Hence, we can conclude that, considering our increasingly 24 h society, researchers should focus their attention on the strict relationship among LAN, melatonin production blocking, circadian rhythm disruption, and increased risk of cancers and should search for new therapeutic strategies.
